# Molecular profile and its clinical impact of IDH1 mutated versus IDH1 wild type intrahepatic cholangiocarcinoma

**DOI:** 10.1038/s41598-022-22543-z

**Published:** 2022-11-05

**Authors:** Margherita Rimini, Carles Fabregat-Franco, Valentina Burgio, Sara Lonardi, Monica Niger, Mario Scartozzi, Ilario Giovanni Rapposelli, Giuseppe Aprile, Francesca Ratti, Federica Pedica, Helena Verdaguer, Mario Rizzato, Federico Nichetti, Eleonora Lai, Alessandro Cappetta, Teresa Macarulla, Matteo Fassan, Filippo De Braud, Andrea Pretta, Francesca Simionato, Francesco De Cobelli, Luca Aldrighetti, Lorenzo Fornaro, Stefano Cascinu, Andrea Casadei-Gardini

**Affiliations:** 1 Department of Medical Oncology, IRCCS San Raffaele Scientific Institute Hospital, Vita-Salute San Raffaele University, Via Olgettina n. 60, Milan, Italy; 2 Gastrointestinal Cancer Unit, Vall d’Hebron University Hospital, Vall d’Hebron Institute of Oncology (VHIO), Barcelona, Spain; 3 Oncology Unit 3, Veneto Institute of Oncology-IRCCS, Padua, Italy; 4 Medical Oncology Department, Fondazione IRCCS Istituto Nazionale dei Tumori di Milano, Milan, Italy; 5 Medical Oncology, University and University Hospital, Cagliari, Italy; 6 Department of Medical Oncology, IRCCS Istituto Romagnolo per lo Studio dei Tumori (IRST) “Dino Amadori”, 47014 Meldola, Italy; 7 Department of Oncology, San Bortolo General Hospital, Azienda ULSS8 Berica, Vicenza, Italy; 8 Hepatobiliary Surgery Division, Liver Center, IRCCS San Raffaele Scientific Institute, Vita-Salute San Raffaele University, 20132 Milan, Italy; 9 Pathology Unit, Department of Experimental Oncology, IRCCS San Raffaele Scientific Institute, 20132 Milan, Italy; 10 Oncology Unit 1, Veneto Institute of Oncology-IRCCS, Padua, Italy; 11 Surgical Pathology Unit, Department of Medicine (DIMED), University of Padua, Padua, Italy; 12 Veneto Institute of Oncology-IRCCS, Padua, Italy; 13 Department of Oncology and Hemato-Oncology, University of Milan, Milan, Italy; 14 School of Medicine, Vita-Salute San Raffaele University, 20132 Milan, Italy; 15 Department of Oncology, IRCCS San Raffaele Scientific Institute Hospital, Vita-Salute San Raffaele University, Milan, Italy

**Keywords:** Cancer genomics, Gastrointestinal cancer

## Abstract

*IDH1*-mutated cholangiocarcinomas (CCAs) are an interesting group of neoplasia with particular behavior and therapeutic implications. The aim of the present work is to highlight the differences characterizing *IDH1*m and *IDH1*wt CCAs in terms of genomic landscape. 284 patients with iCCA treated for resectable, locally advanced or metastatic disease were selected and studied with the FOUNDATION Cdx technology. A comparative genomic analysis and survival analyses for the most relevant altered genes were performed between *IDH1*m and *IDH1*wt patients. Overall, 125 patients were *IDH1*m and 122 *IDH1*wt. *IDH1*m patients showed higher mutation rates compared to *IDH1*wt in *CDKN2B* and lower mutation rates in several genes including *TP53*, *FGFR2*, *BRCA2*, *ATM*, *MAP3K1*, *NOTCH2*, *ZNF703*, *CCND1*, *NBN*, *NF1*, *MAP3KI3*, and *RAD21*. At the survival analysis, *IDH1*m and *IDH1*wt patients showed no statistically differences in terms of survival outcomes, but a trend in favor of *IDH1wt* patients was observed. Differences in prognostic values of the most common altered genes were reported. In surgical setting, in *IDH1*m group the presence of *CDKN2A* and *CDKN2B* mutations negatively impact DFS, whereas the presence of *CDKN2A, CDKN2B*, and *PBRM1* mutations negatively impact OS. In advanced setting, in the *IDH1*m group, the presence of *KRAS/NRAS* and *TP53* mutations negatively impact PFS, whereas the presence of *TP53* and *PIK3CA* mutations negatively impact OS; in the *IDH1wt* group, only the presence of *MTAP* mutation negatively impact PFS, whereas the presence of *TP53* mutation negatively impact OS. We highlighted several molecular differences with distinct prognostic implications between *IDH1*m and *IDH1*wt patients.

## Introduction

Cholangiocarcinoma (CCA) represents a heterogeneous group of malignancies that arises from biliary epithelium, and it is generally regarded as a rare tumor in Western countries^1^. Nevertheless, over the last 15 years its incidence has steadily increased worldwide, and now it represents the second most common type of primary malignancy in the liver (15–20% of cases) after hepatocellular carcinoma^2,3^. Nowadays, platinum-based chemotherapy constitutes the backbone treatment of advanced and metastatic setting, but prognosis remains dismal, with a five-year survival rate of about 2% for stage IV^4^. The unsatisfactory results obtained could be related to some intrinsic characteristics of CCA, and mainly to a general incomprehension of its underlying molecular pathways. Indeed, all the previous clinical trials considered CCA as a whole group of diseases without considering the molecular heterogeneity, thus hindering the development of the optimal therapy aimed at the specific type of biliary tract cancer. Starting from these premises, a better understanding of the biological pathways underlying the carcinogenesis in CCA and an individual characterization of these tumors at the genomic, epigenetic and molecular levels has turned to be an urgent need. Recent advances in technical innovations in high-throughput molecular analysis have led to the discover of new potential therapeutic targets, including tumor suppressor genes involved in DNA damage repair pathway, kinases such as FGFR1, FGFR2, FGFR3, PIK3CA, ALK, EGFR, ERBB2, BRAF and AKT3, and oncogenes such as *CCND3, MDM2* and, notably, *IDH1* and *IDH2*^5^.

*IDH1*-mutated CCAs constitute a group of neoplasms of particular interest in the biliary tract cancer field, due to a particular behavior and therapeutic implications. *IDH* genes encode for three different IDH enzymes, which are known to play an important role in the Krebs cycle and in cell metabolism^6,7^. In physiological conditions, IDH1 and IDH2 enzymes are involved in a two-step reaction which converts the isocitrate (ICT) in α-ketoglutarate (α-KG) by reducing the NADP+ in NADPH^8–10^. Given the involvement of IDH1 and IDH2 in cell metabolism, gain-of-function mutations of these genes lead to the accumulation of the oncometabolite 2-hydroxyglutarate (2-HG), as consequence of the neomorphic ability to convert α-KG into 2-HG^11^. 2-HG does not participate in normal metabolic processes but instead interferes with theα-KG -dependent reactions, thus resulting in DNA and histone hypermethylation, genetic instability, hypoxia gene signature activation, oxidative stress and alteration of the mTOR pathway and the mitochondrial electron transport chain^12^. *IDH1/2* mutated forms are mostly absent in perihilar CCA (pCCA) and distal CCA (dCCA)^12^, whereas represent the 25% of intrahepatic CCA (iCCA) cases, with some differences depending on the geographical location^12^.

The discovery of mutations in *IDH* genes (*IDH1* and *IDH2*) has revolutionized the therapeutic approaches and opened a new research way focused on possible targeted therapies capable of inhibiting the aberrant activity of the mutated isoforms. Nowadays, a number of *IDH1* inhibitors are under investigations^13^; among these, AG-120 (Ivosidenib) received the FDA approval in advanced and metastatic CCA patients with IDH1 mutations due to the promising results of the randomized phase III trial ClarIDHy^14^.

Recently, high-throughput genomic sequencing techniques permitted to highlight the heterogeneous genomic scenario of iCCA harboring *IDH1* mutations^12,15–17^. Nevertheless, these data are still far to be conclusive, since a significant heterogeneity between the cohorts and differences in inclusion criteria have to be considered.

The aim of the present work is to highlight the molecular differences in IDH1 mutated versus IDH1wt CCAs, with a special focus on the most relevant genomic alterations and their prognostic value in both CCA patients receiving a surgical intervention and those treated with systemic therapy.

## Material and methods

### Patients’ enrollment and sample collection

For this study, we selected 284 patients with iCCA treated for resectable, locally advanced or metastatic disease in six Italian institutions and one Spanish institute from January 2013 to March 2021. The sample included two different cohorts of patients: the first one included patients diagnosed at local stage who received radical surgery, and the second one included patients who relapsed after surgery or who were diagnosed at locally advanced or metastatic stages and judged to be candidate to receive exclusively systemic treatment. All patients were reviewed to confirm the pathologic diagnosis of ICC and evaluated with a chest-abdomen computed tomography (CT) according to the 8th edition 2017 AJCC staging system. After exclusion of 37 patients for lack of clinic-pathological and genomic information (including 10 cases lost to follow-up, 15 cases who lack clinical information and 12 patients who lack genomic information), 247 patients were eventually used for comparison of clinical, molecular and genomic characteristics and survival analysis (Supplementary Figure). Formalin-fixed paraffin-embedded (FFPE) samples and hematoxylin–eosin staining slides of the 247 patients (surgical specimens for patients who underwent surgery and biopsy specimens for patients who did not undergo surgery during their clinical history) were collected from Pathology Department of each single institutions. A full histopathologic review was performed by an expert gastrointestinal pathologist. Genic analysis of the primary tumors was performed by the FOUNDATION Cdx technology.

### Clinical data

Clinical data including the patients’ gender, age, Eastern Cooperative Oncology Group (ECOG) Performance Status, kind of treatment received (surgical versus systemic, and type of systemic therapy) and pathological data, including surgical records when available, primary tumor location, histological grading and TNM stage according to the 8th edition 2017 AJCC staging system were carefully collected at the baseline, and used for analysis. Response to systemic treatment was assessed using RECIST criteria. For patients receiving a radical surgery, the follow-up was planned after 4 weeks from the intervention, and then each three months by performing a chest-abdomen CT-scan, laboratory tests including the Ca 19.9 and CEA blood-levels and clinical examination, until the evidence of relapsed disease. For patients receiving systemic treatment, response was assessed every 8–12 weeks by performing a chest-abdomen CT-scan, according to each institution’s clinical protocol. Patients receiving systemic therapy were treated according to the physician choice. For patients treated surgically, disease free survival (DFS) and overall survival (OS) from surgery were calculated. DFS was measured from the date of surgery to the date of first recurrence or last follow-up, whereas OS from surgery was defined as the interval between the date of surgery and the date of death or last follow-up. For patients diagnosed for a locally advanced or metastatic disease, who were stained not eligible for surgery, progression free survival (PFS) and OS from the first line treatment were calculated. PFS was measured from the date of the start of the first line therapy to the date of first recurrence or last follow-up. OS from the first line treatment was defined as the interval between the date of the first-line start and the date of death or last follow-up.

### Identification of genomic alterations

FFPE tumor tissues containing at least 20% of tumor cells were collected from patients for genomic analysis detection by the NGS-based FoundationOne (FoundationOneR, Foundation Medicine Inc., MA, USA) gene panel. Identified alterations included base substitutions, insertions/delections (1–40 bp), copy number alterations-amplifications (ploidy < 4, amplification with copy number ≥ 8), copy number alterations-delections (ploidy < 4, homozygous delections), rearrangements and microsatellite status (determined by assessing indel characteristics at 114 homopolymer repeat loci in or near the targeted gene regions of the FoundationOne test).. The Foundation Medicine assay used was designed to analyze all genes know to be somatically altered in human solid tumors that are validated targets for therapy, either approved or in clinical trials, and/or that are unambiguous drivers of oncogenesis based on current knowledge. The assay employed a single DNA extraction method from routine FFPE biopsy or surgical resection specimens; 50–1000 ng o DNA underwent whole-genome shotgun library construction and hybridization-based capture of all coding exons from 309 cancer-related genes, one promotor region, one non-coding (ncRNA), and selected intronic regions from 34 commonly rearranged genes, 21 of which also included the coding exons. In total, the assay detects alterations in a total of 324 genes. Using the Illumina® HiSeq 4000 platform, hybrid capture–selected libraries were sequenced to high uniform depth (targeting > 500× median coverage with > 99% of exons at coverage > 100×). Sequence data were then processed using a customized analysis pipeline designed to detect all classes of genomic alterations, including base substitutions, indels, copy number alterations (amplifications and homozygous gene deletions), and select genomic rearrangements (e.g., gene fusions)^18^.

A descriptive analysis of the molecular landscape in the entire sample and in the two groups of patients was performed.

### Statistical analysis

Categorical variables were presented as totals and frequencies, then evaluated by Chi-squared test of Fisher exact test, as appropriate. Continuous variables were described as means with standard deviations or medians with ranges, and compared with T test. The genomic alterations present in ≥ 5% of the entire sample were considered for the analysis of distribution of genomic alterations in the two groups of patients (*IDH1*m versus *IDH1*wt). The distribution analysis was performed by Fisher exact test. For the *IDH1*m and *IDH1*wt patients, correlative analyses between genetic alterations and survival outcomes were performed. DFS and OS from surgery, as well as PFS and OS from first line therapy were calculated by Kaplan–Meier method, and assessed by log-rank test for univariate analysis. The results were recorded as hazard ratios (HR) and 95% confidence intervals (CIs). A two-tailed P values less than 0.05 was considered statistically significant. DFS and OS from surgery as well as PFS and OS from the start of the first line treatment were estimated by the Kaplan–Meier method and curves were compared by the log-rank test. A p value < 0.05 was considered statistically significant. A MedCalc package (MedCalc® version 16.8.4) was used for statistical analysis. For the comparative genomic analysis as well as for the survival analysis of the sample who underwent to surgery and the sample who received first line chemotherapy, we considered only the gene alterations which were present at least in 7% of the whole cohort of patients.

### Ethical approval

The study was conducted in accordance with the Declaration of Helsinki and the protocol was approved by the Ethics Committee of San Raffaele Hospital with number of registry: 113/INT/2021. Under the condition of retrospective archival tissue collection and patients’ data anonymization, our study was exempted from the acquisition of informed consent from patients by the institutional review board.

### Institutional review board statement

The Ethical Review Board of each Institutional Hospital approved the present study. This study was performed in line with the principles of the Declaration of Helsinki.

### Informed consent statement

Written informed consent for treatment was obtained for all patients.

## Results

### Clinical characteristics in intrahepatic cholangiocarcinoma patients

Overall, 247 consecutive iCCA patients were retrospectively analyzed. 125 patients were *IDH1*m and 122 *IDH1*wt. 128/247 patients received surgical treatment. By considering the two populations, *IDH1*m and *IDH1*wt patients, no significative differences were found in terms of clinic-pathological characteristics, except for the gender (female 65.60% vs 40.98% in *IDH1*m group and *IDH1*wt group, respectively; p = 0.000126). The median age at diagnosis was 59^28^ in IDH1m group compared to 62 (33–83) in *IDH1*wt group. At the baseline, 7% and 2% of patients were diagnosed of CCA at stage I, whereas 76% and 43% were diagnosed at more advanced stages (II, III and IV), in the *IDH1*m and *IDH1*wt groups, respectively (p = 0.3304). At the start of the first-line therapy, 69/125 (55%) and 60/122 (49%) of patients presented an ECOG PS of 0 in *IDH1*m and *IDH1*wt groups of patients, respectively (p = 0.6587). In the *IDH1*m group, the 46% received surgical intervention with radical intention and 86% were treated with systemic therapy during their oncologic history; in the *IDH1*wt group of patients, 58% received surgical intervention with radical intention and 82% were treated with systemic therapy during their oncologic history. Finally, 90/125 (72%) of *IDH1*m patients and 75/122 (61.5%) of *IDH1*wt patients received the first line standard of care cisplatin plus gemcitabine, whereas 17/125 (13%) and 25/122 (20.5%) received other regimens in *IDH1*m and *IDH1*wt groups of patients, respectively (p = 0.120798). No patient received Ivosidenib or another IDH1 inhibitor as first line treatment in our sample (Table 1).

**Table 1:** Patients’ characteristics according to the IDH1 status.

	IDH1 mutated (N = 125) N (%)	IDH1 wild type (N = 122) N (%)	P
**Gender**			
Male	43 (34)	72 (60)	**0.000126**
Female	82 (66)	50 (40)	
**Age**			
≥ 70	25 (20)	30 (24.5)	0.445180
< 70	100 (80)	92 (75.5)	
**Grading**			
G1	2 (1.5)	5 (4)	0.5261
G2	16 (13)	12 (10)	
G3	26 (21.5)	22 (18)	
NA	80 (64)	83 (68)	
**Stage disease**			
I	9 (7)	3 (2)	0.3304
II	12 (10)	11 (9)	
III	17 (14)	12 (10)	
IV	65 (52)	29 (24)	
NA	22 (18)	67 (55)	
**ECOG PS**			
0	69 (55)	60 (49)	0.6587
1	25 (20)	26 (21)	
≥ 2	7 (6)	4 (3)	
NA	24 (19)	32 (26)	
**Primary tumor resected**			
Yes	57 (46)	71 (58)	0.056276
No	68 (54)	51 (42)	
**Systemic therapy for advanced disease**			
Yes	107 (86)	100 (82)	0.4040
No	14 (11)	18 (15)	
NA	4 (3)	4 (3)	
**First line therapy**			
Cisplatin/gemcitabine	90 (72)	75(61.5)	0.120798
Others	17 (13)	25 (20.5)	

Significant values are in bold.

### Genomic alterations in intrahepatic cholangiocarcinoma patients

**Figure 1:**
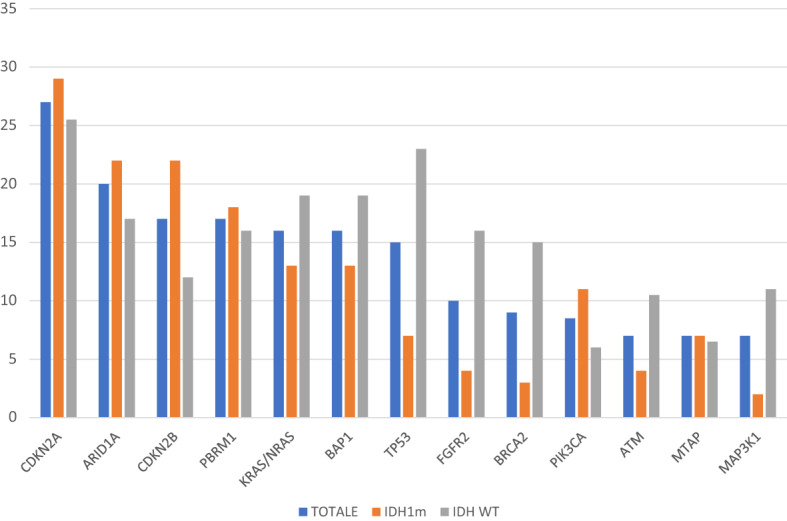
Most common altered genes in the whole sample, in IDH1m patients and in IDH1wt patients.

We first annotated alterations to specific genes. Overall, genomic sequencing performed by FoundatioOne assays identified a total of 1446 genomic alterations in the entire sample which involved 262 genes, with a mean of 4.65 alterations per gene (range 0–66). All the samples presented at least one genomic alteration, with a median of genomic alterations for patients of 7.21 (range 1–44). The most common genomic alterations were found in *CDKN2A* (27%), *ARID1A* (20%), *CDKN2B* (17%), *PBRM1* (17%), *KRAS/NRAS* (16%), *BAP1* (16%), *TP53* (15%), *FGFR2* (10%), *BRCA2* (9%), *PIK3CA* (8.5%), *ATM* (7%), *MTAP* (7%) and *MAP3K1* (7%) (Fig. 1).

Mutations of all remaining genes were detected less than 7% of the entire sample, and 15% of all the analyzed genes were mutated once in a single sample, including *KIT, PIK3CG, NTRK2, GATA4, BCL2, FANCC, FGFR1, MAP2K1, MAPK1, RAD51* and *RAD52*.

### Genomic alterations in IDH1m versus IDH1wt patients

A comparative analysis of the *IDH1*m group versus the *IDH1*wt group of patients highlighted several differences in terms of mutations distribution and molecular landscape. Overall, *IDH1*m CCA showed a lower incidence of detected mutations compared to *IDH1*wt CCA. If we focus on concrete gene alterations, *IDH1*m patients showed higher mutation rate compared to *IDH1*wt in *CDKN2B* (22% Vs 12%, p = 0.04) and lower mutation rate in *TP53* (7% versus 23%, p = 0.0006), *FGFR2* (4% versus 16%, p = 0.0013), *BRCA2* (3% versus 15%, p = 0.0015), *ATM* (4% versus 10.5%, p = 0.047) *MAP3K1* (2% versus 11%, p = 0.0053), *NOTCH2* (1.5% versus 10%, p = 0.005), *ZNF703* (1.5% versus 7%, p = 0.033), *CCND1* (1% versus 7%, p = 0.01), *NBN* (0% versus 7%, p = 0.0015), *NF1* (0% versus 6.5%, p = 0.003), *MAP3KI3* (1% versus 6%, p = 0.034), *RAD21* (0% versus 6.5%, p = 0.003), *ESR1* (0% versus 6%, p = 0.007), *GATA6* (0% versus 6%, p = 0.007), *MYC* (o% versus 5%, p = 0.014), *AXIN1* (0% versus 5%, p = 0.014), *TSC2* (0% versus 5%, p = 0.014), *PARP2* (0% versus 5%, p = 0.014), *WHSC1L1* (0% versus 5%, p = 0.014), *ERBB3* (0% versus 5%, p = 0.014), *ALK* (0% versus 4%, p = 0.03), *HSD3B1* (0% versus 4%, p = 0.03), *PARK2* (0% versus 4%, p = 0.03), *CTN1* (0% versus 4%, p = 0.03), *MITF* (0% versus 4%, p = 0.03), *RICTOR* (0% versus 4%, p = 0.03), *BCOR* (0% versus 4%, p = 0.03), *NTRK1* (0% versus 4%, p = 0.03), *EPHA3* (0% versus 4%, p = 0.03) and *DOT1L* (0% versus 4%, p = 0.03) (Table 2).

**Table 2:** Genomic alterations in whole sample and according to the IDH1 status.

GENE	Total, N = 247 (%)	IDHm, N = 125 (%)	IDH WT, N = 122 (%)	P
CDKN2A	66 (27)	36 (29)	30 (25.5)	0.475252
ARID1A	49 (20)	28 (22)	21 (17)	0.340628
CDKN2B	43 (17)	28 (22)	15 (12)	**0.043975**
PBRM1	43 (17)	23 (18)	20 (16)	0.738452
KRAS/NRS	39 (16)	16 (13)	23 (19)	0.223471
BAP1	39 (16)	16 (13)	23 (19)	0.223471
TP53	37 (15)	9 (7)	28 (23)	**0.000598**
FGFR2	25 (10)	5 (4)	20 (16)	**0.001322**
BRCA2	22 (9)	4 (3)	18 (15)	**0.001489**
PIK3CA	21 (8.5)	14 (11)	7 (6)	0.170611
ATM	18 (7)	5 (4)	13 (10.5)	**0.046808**
MTAP	17 (7)	9 (7)	8 (6.5)	1.000000
MAP3K1	17 (7)	3 (2)	14 (11)	**0.005268**
MDM2	16 (6)	6 (5)	10 (8)	0.311262
IRS2	15 (6)	4 (3)	11 (9)	0.065374
MED12	15 (6)	4 (3)	11 (9)	0.065374
NOTCH2	14 (6)	2 (1.5)	12 (10)	**0.005448**
MUTYH	14 (6)	7 (5.5)	7 (6)	1.000000
PIK3C2B	13 (5)	5 (4)	8 (6.5)	0.406554
MCL1	13 (5)	6 (5)	7 (6)	0.782716
MLL2	12 (5)	3 (2)	9 (7)	0.081463
FGF19	12 (5)	2 (1.5)	10 (8)	0.018283
ERCC4	11 (4)	4 (3)	7 (6)	0.371923
DNMT3A	11 (4)	6 (5)	5 (4)	1.000000
ZNF703	11 (4)	2 (1.5)	9 (7)	**0.032749**
GS	10 (4)	3 (2)	7 (6)	0.212393
ZNF217	10 (4)	2 (1.5)	8 (6.5)	0.057665
ATR	10 (4)	3 (2)	7 (6)	0.212393
CCND1	10 (4)	1 (1)	9 (7)	**0.009500**
NBN	9 (3.5)	0 (0)	9 (7)	**0.001499**
CREBBP	9 (3.5)	3 (2)	6 (5)	0.329773
PTEN	8 (3)	4 (3)	4 (3)	1.000000
KDM5C	8 (3)	4 (3)	4 (3)	1.000000
KDR	8 (3)	5 (4)	3 (2)	0.722201
NOTCH1	8 (3)	3 (2)	5 (4)	0.496227
APC	8 (3)	4 (3)	4 (3)	1.000000
NF1	8 (3)	0 (0)	8 (6.5)	**0.003142**
MAP3KI3	8 (3)	1 (1)	7 (6)	**0.034306**
TERT	8 (3)	2 (1.5)	6 (5)	0.168288
RAD21	8 (3)	0 (0)	8 (6.5)	**0.003142**
BRCA1	7 (3)	1 (1)	6 (5)	0.063833
ERRF1	7 (3)	5 (4)	2 (1.5)	0.446622
RNF43	7 (3)	1 (1)	6 (5)	0.063833
ESR1	7 (3)	0 (0)	7 (6)	**0.006558**
SNCAIP	7 (3)	2 (1.5)	5 (4)	0.277142
IGF1R	7 (3)	1 (1)	6 (5)	0.063833
GATA6	7 (3)	0 (0)	7 (6)	**0.006558**
ERBB4	7 (3)	2 (1.5)	5 (4)	0.277142
PTPN11	7 (3)	3 (2)	4 (3)	0.719867
IKBKE	7 (3)	1 (1)	6 (5)	0.063833
SPEN	7 (3)	3 (2)	4 (3)	0.719867
FGF3	7 (3)	1 (1)	6 (5)	0.063833
FGF4	7 (3)	1 (1)	6 (5)	0.063833
MSH3	7 (3)	2 (1.5)	5 (4)	0.277142
CIC	7 (3)	2 (1.5)	5 (4)	0.277142
KMT2D	7 (3)	2 (1.5)	5 (4)	0.277142
TSC1	6 (2.5)	4 (3)	2 (1.5)	0.683784
FGFR4	6 (2.5)	4 (3)	2 (1.5)	0.683784
NOTCH3	6 (2.5)	2 (1.5)	4 (3)	0.442670
EGFR	6 (2.5)	5 (4)	1 (1)	0.213220
POLD1	6 (2.5)	4 (3)	2 (1.5)	0.683784
ROS1	6 (2.5)	1 (1)	5 (4)	0.116774
ERBB3	6 (2.5)	0 (0)	6 (5)	0.013625
JAK3	6 (2.5)	1 (1)	5 (4)	0.116774
CDK6	6 (2.5)	1 (1)	5 (4)	0.116774
EPHB4	6 (2.5)	1 (1)	5 (4)	0.116774
ABL1	6 (2.5)	1 (1)	5 (4)	0.116774
MYC	6 (2.5)	0 (0)	6 (5)	**0.013625**
ASXL1	6 (2.5)	2 (1.5)	4 (3)	0.442670
AXIN1	6 (2.5)	0 (0)	6 (5)	**0.013625**
PTCH1	6 (2.5)	2 (1.5)	4 (3)	0.442670
TSC2	6 (2.5)	0 (0)	6 (5)	**0.013625**
PARP2	6 (2.5)	0 (0)	6 (5)	**0.013625**
PDGFRB	6 (2.5)	1 (1)	5 (4)	0.116774
WHSC1L1	6 (2.5)	0 (0)	6 (5)	**0.013625**
PARP1	6 (2.5)	2 (1.5)	4 (3)	0.442670
RB1	6 (2.5)	2 (1.5)	4 (3)	0.442670
PIK3C2G	5 (2)	4 (3)	1 (1)	0.370075
MLL	5 (2)	3 (2)	2 (1.5)	1.000000
BRD4	5 (2)	1 (1)	4 (3)	0.209332
RET	5 (2)	1 (1)	4 (3)	0.209332
FLT3	5 (2)	1 (1)	4 (3)	0.209332
ALK	5 (2)	0 (0)	5 (4)	**0.028181**
HSD3B1	5 (2)	0 (0)	5 (4)	**0.028181**
PARK2	5 (2)	0 (0)	5 (4)	**0.028181**
CTN1	5 (2)	0 (0)	5 (4)	**0.028181**
MITF	5 (2)	0 (0)	5 (4)	**0.028181**
RICTOR	5 (2)	0 (0)	5 (4)	**0.028181**
MSH6	5 (2)	1 (1)	4 (3)	0.209332
BCOR	5 (2)	0 (0)	5 (4)	**0.028181**
NTRK1	5 (2)	0 (0)	5 (4)	**0.028181**
EPHA3	5 (2)	0 (0)	5 (4)	**0.028181**
DOT1L	5 (2)	0 (0)	5 (4)	**0.028181**
MDM4	5 (2)	2 (1.5)	3 (2)	0.681149
RAD54L	5 (2)	2 (1.5)	3 (2)	0.681149
RAF1	5 (2)	2 (1.5)	3 (2)	0.681149

Significant values are in bold.

### Targetable alterations

**Figure 2:**
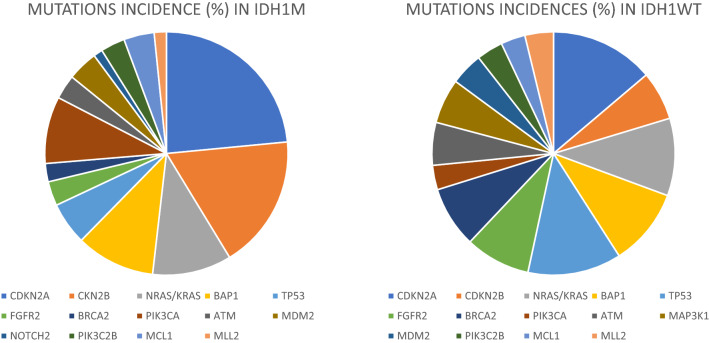
Mutations’ incidence in IDH1m patients and in IDH1wt patients.

We identified 22 frequently (≥ 5%) mutated genes identified in the whole sample (Table 2), where 15 were highlighted to be actionable according to the TARGET database by the Broad Institute (http://archive.broadinstitute.org/cancer/cga/target), including: *CDKN2A, CDKN2B, NRAS/KRAS, BAP1, TP53, FGFR2, BRCA2, PIK3CA, ATM, MDM2, PIK3C2B, NOTCH2, MCL1, MLL2*. By comparing the two scenarios, those of the *IDH1*m patients and those of the *IDH1*wt patients, several differences in terms of incidence of targetable mutations has been highlighted (Fig. 2).

### Survival analysis according to the genomic landscape

#### Resected patients

The survival analysis for DFS and OS from surgery was performed on the sample of resected patients (N = 128).

At the univariate analysis, *IDH1*m and *IDH1*wt patients showed no statistically differences in terms of DFS and OS from surgery (p = 0,6156, p = 0,2645; respectively). Nevertheless, a tendence toward a better OS was highlighted for *IDH1*wt patients compared *IDH1*m patients.

**Figure 3:**
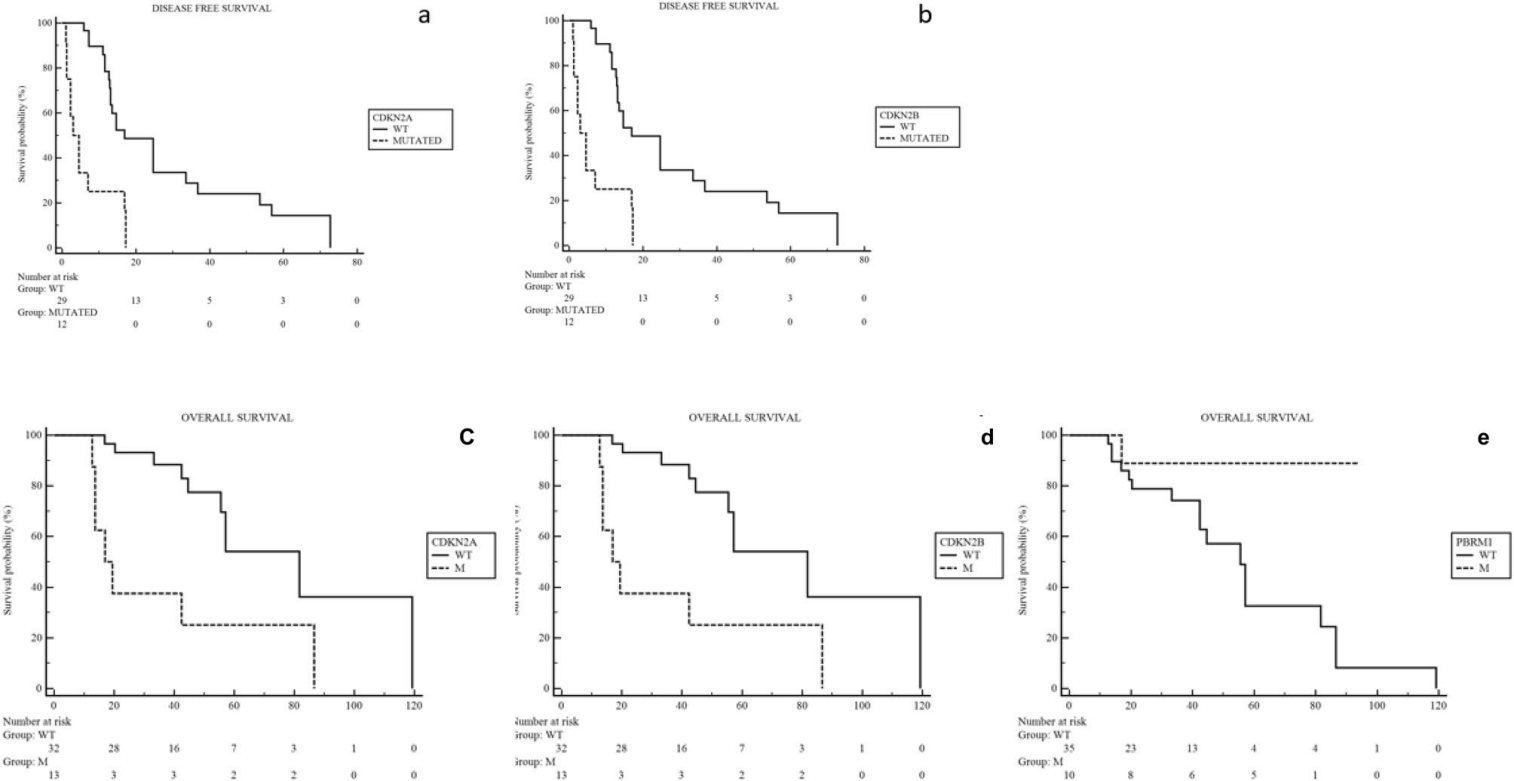
Kaplan Meyer curves of DFS and OS from surgery according to the altered genes with prognostic impact.

At the univariate analysis for DFS from surgery conducted for the most commonly altered genes in our sample, in the *IDH1*m group the presence of *CDKN2A* and *CDKN2B* mutations were highlighted to have a negative prognostic impact (*CDKN2A* HR 3.78, 95% CI 1.37–10.41, p = 0.0001; *CDKN2B* HR 3.46, 95% CI 1.68–10.28, p = 0.0004) (Fig. 3a,b). On the other hand, no one gene showed to affect prognosis in terms of DFS from surgery in the *IDH1*wt group of patients (Supplementary Table 1).

At the univariate analysis for OS from surgery conducted for the most commonly altered genes in our sample, in the *IDH1*m group of patients the presence of *CDKN2A, CDKN2B*, and *PBRM1* mutations were highlighted to have a negative prognostic impact (*CDKN2A* HR 3.20, 95% CI 1.01–10.14, p = 0.0096; *CDKN2B* HR 3.20, 95% CI 1.01–10.14, p = 0.0096; *PBRM1* HR 6.61, 95% CI 2.36–18.50, p = 0.04) (Fig. 3c–e). On the other hand, no one gene showed to affect prognosis in terms of OS from the surgical intervention in the *IDH1*wt group of patients (Supplementary Table 1). After adjustment for the clinical covariates known to be related to prognosis in this setting of patients (Stage disease and ECOG Performance Status), alterations in CDKN2A and MTAP were confirmed to be negative prognostic factor for DFS in this cohort of patients.

#### Patients treated with systemic therapy

The analysis for PFS and OS from the start of first line therapy was performed on the sample of patients receiving systemic treatments (N = 207).

At the univariate analysis, *IDH1*m and *IDH1*wt patients showed no statistically differences in terms of OS and PFS from the first-line treatment (p = 0.1179, p = 0.6203; respectively). Nevertheless, a tendence toward a better OS was highlighted for *IDH1*wt patients compared *IDH1*m patients.

**Figure 4:**
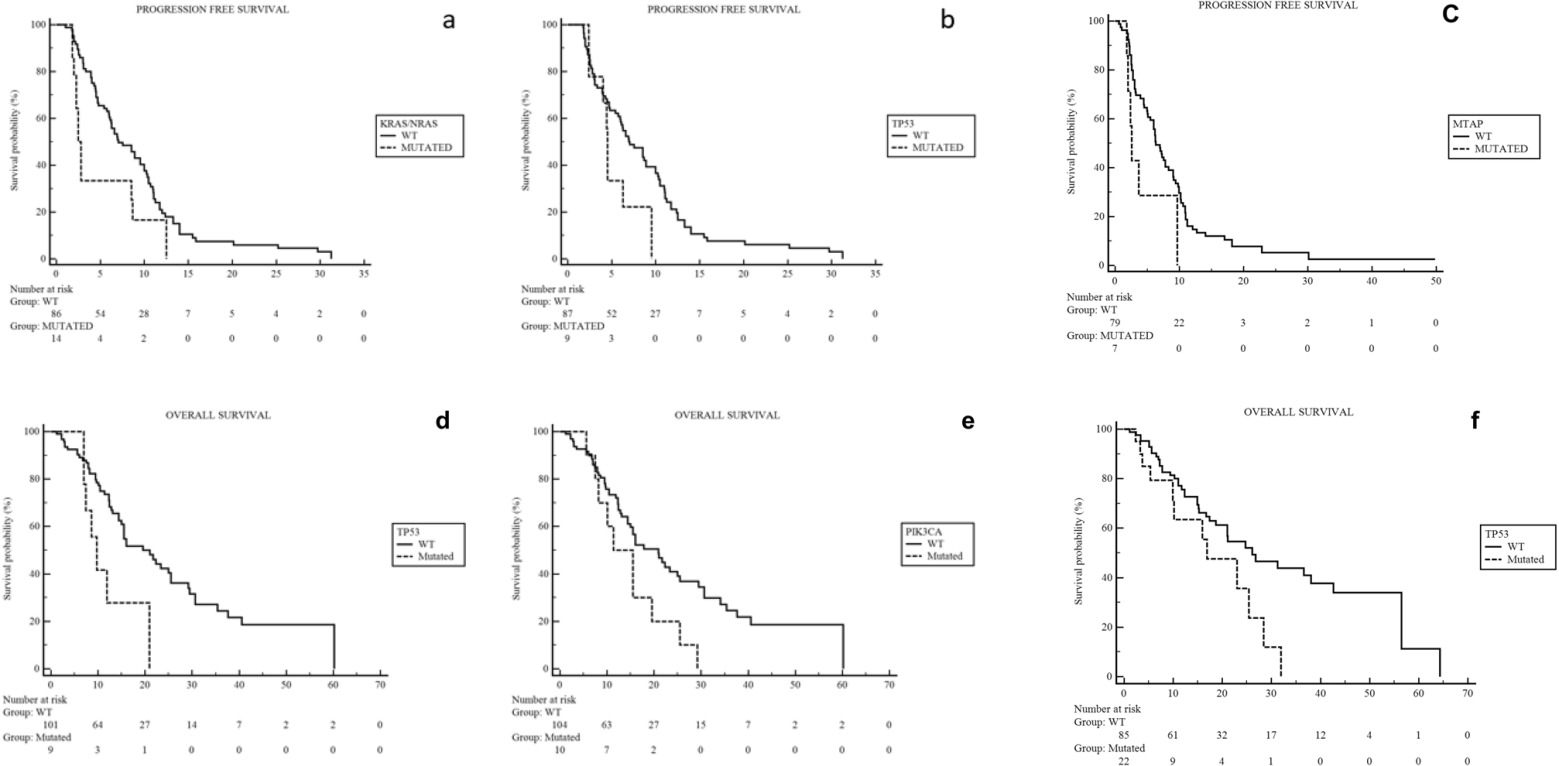
Kaplan Meyer curves of PFS and OS from first line therapy according to the altered genes with prognostic impact.

At the univariate analysis for PFS from the first line therapy conducted for the most commonly altered genes in our sample, in the *IDH1*m group of patients, the presence of *KRAS/NRAS* and *TP53* mutations were highlighted to negatively affect prognosis (*KRAS/NRAS* HR 2.06, 95% CI 0.94–4.51, p = 0.0136; TP53 HR 2.05, 95% CI 0.80–5.22, p = 0.0377) (Fig. 4a,b).

On the other hand, in the *IDH1*wt group of patients, only the presence of *MTAP* mutation was highlighted to have a negative prognostic impact on PFS from the first-line treatment (HR 3.40, 95% CI 1.11–10.46, p = 0.0327) (Fig. 4c) (Supplementary Table 2).

At the univariate analysis for OS from the first line therapy conducted for the most commonly altered genes in our sample, *IDH1*m and *IDH1*wt patients showed a different behavior. In particular, in the *IDH1*m group of patients, the presence of *TP53* and *PIK3CA* mutations were highlighted to negatively impact the prognosis (*TP53* HR 4.39, 95% CI 1.34–14.41, p = 0.0146; *PIK3CA* HR 2.67, 95% CI 1.09–6.57, p = 0.0320) (Fig. 4d,e).

On the other hand, in the *IDH1*wt group, only the presence of *TP53* mutation was highlighted to have a negative prognostic impact in terms of OS from the first-line treatment (HR 1.97 95% CI 0.88–4.39, p = 0.0355) (Fig. 4f) (Supplementary Table 2). After adjustment for the clinical covariates known to be related to prognosis in this setting of patients (Stage disease and ECOG Performance Status), alterations in TP53 were confirmed to be negative prognostic factors for PFS and OS in this cohort of patients.

## Discussion

The present work has the merit to be a comprehensive genomic analysis conducted on a large sample of iCCA patients, which include both *IDH1*m and *IDH1*wt cases, thus highlighting differences in terms of molecular profile between these two groups of patients. In the whole sample, our analysis reported a high incidence of genomic alterations in *CDKN2A, ARID1A, CDKN2B, PBRM1, KRAS/NRAS, BAP1, TP53, FGFR2*, which were highlighted in ≥ 10% of the entire sample of patients. Several studies recently analyzed molecular landscape of CCA by integrate genomic, transcriptomic and epigenomic data, some of them with a special focus on iCCA patients. Lowery et al. reported the genome profiling of CCA patients, including 152 iCCA and 43 eCCA, from Caucasian (89.2%, 174/195), Asian (7.1%, 14/195) and African American (3.6%, 7/195) patients and found that the most common mutations were *IDH1, TP53, ARID1A, BAP1, KRAS, PBKM1, SMAD4 and ATM*^15^. In the recent analysis from Jiang and collaborators, the most frequent mutated genes found in Chinese CCA patients were *TP53* (41.27%, 26/63), *KRAS* (31.75%, 20/63), *ARID1A* and *IDH1* (15.87%, 10/63, for both), *SMAD4* (14.29%, 9/63), *FGFR2* and *BAP1* (12.70%, 8/63, for both) and *CDKN2A* (11.11%, 7/63)^19^. Recently, a bi-institutional study on 412 iCCA patients revealed as most common mutated genes *IDH1* (20%), *ARID1A* (20%), *TP53* (17%), *CDKN2A* (15%), *BAP1* (15%), *FGFR2* (15%), *PBRM1* (12%) and *KRAS* (10%)^19^. Notably, these latter results were particularly similar to those highlighted in our analysis, with the exception of *CDKN2A*, which was more frequently mutated in our cohort of patients.

The differences reported between our analysis and the previous ones in terms of mutations frequencies could be explained by referring to the differences of the cohorts, as well as to the inclusion criteria. Indeed, our analysis included exclusively European iCCA patients from two different countries (Italy and Spain), whereas several previous experiences were conducted on mixed population which included Asiatic patients, or samples exclusively composed by Asiatic patients, which have been previously highlighted to carry different mutational profiles compared western populations^19^. Moreover, due to the rarity of the disease, the previous studies were not focused on *IDH1*m CCA patients, and the sample size of *IDH1*m patients in the cohorts were too small to characterize this subtype of iCCA.

By performing a comparative analysis, we highlighted two different molecular profiles for *IDH1*m and *IDH1*wt patients. More specifically, *IDH1*m samples showed a lower incidence of genomic alterations compared with *IDH1*wt samples, and were highlighted to be enriched in *CDKN2A* (29%), *ARID1A* (22%), *CDKN2B* (22%), *PBRM1* (18%), *NRAS/KRAS* (13%), *BAP1* (13%), *PIK3CA* (11%), *TP53* (7%), *MTAP* (7%), *MUTYH* (5.5%), *MDM2* (5%), *MCL1* (5%) and *DNMT3A* (5%). If considering the most relevant genomic alterations in our sample, *CDKN2B* was highlighted to be more frequently mutated in *IDH1*m patients, whereas *TP53, FGFR2, BRCA2, ATM, MAP3K1* and *NOTCH2* resulted to be more frequently altered in *IDH1*wt patients. Consistently with these results, in a previous work Farshdifar and collaborators identified an *IDH*m-enriched subtype with distinct molecular features including low expression of chromatin modifiers (also cadherins), elevated expression of mitochondrial genes, and increased mitochondrial DNA copy number^20,21^.

Interestingly, several differences in terms of incidence of targetable mutations according the TARGET database by the Broad Institute (http://archive.broadinstitute.org/cancer/cga/target) have been highlighted between *IDH1*m and *IDH1*wt patients. Nowadays, the identification of targetable mutations is a hot topic in oncologic field, since patients carrying one or more actionable lesions could have broad opportunities for treatment. The identifications of differences in terms of targetable mutations’ incidence between *IDH1*m and *IDH1*wt patients could suggest novel therapeutic strategies which could be investigated in concomitating and/or in sequencing to the recently studied *IDH* inhibitors. In particular, our results could suggest that *IDH1*m patients may benefit from treatments which interfere with the cell cycle, such as *CDK 4/6* inhibitors; on the other hand, *IDH1*wt patients may benefit from *PARP* inhibitors and *FGFR2* inhibitors. Further studies are needed in order to verify our hypothesis, with the hope that new prospective trials investigating the efficacy of personalized target therapies could be designed in the next future for this setting of patients.

Concerning the survival analysis, several interesting considerations could be done. Firstly, in the cohort of patients receiving surgery, the analysis revealed *CDKN2A/B* alterations as negative prognostic factors in terms of DFS and OS from surgery in the subset of patients carrying *IDH1* mutations, whereas no prognostic implication was highlighted in *IDH1*wt patients. Significantly, mutations in CDKN2A were confirmed to be negative prognostic factor in terms of DFS after adjustment for the clinical covariates known to impact prognosis in patients receiving surgery.

Cyclin-dependent kinase (*CDK*) inhibitor 2A (*CDKN2A*) and 2B (*CDKN2B*) are known to play an important role in cell-cycle regulation through inhibition of *CDK4/6*. *CDKN2A/2B* loss or mutation are associated with tumor progression, invasion and metastasis and have been reported in 7–18% of iCCAs^22–24^. Basing on these reports, CDK4/6 inhibitors have been recently tested in monotherapy in patients carrying *CDKN2A* alterations, without the wished results^25^. Previously, Lowery and collaborators showed that alterations in *CDKN2A/B* were associated with reduced survival and time to progression on chemotherapy in patients with locally advanced or metastatic disease^15^. Interestingly, the role of *CDKN2A/B* alterations in CCA is consistent with those found in other onco-hematologic settings, such as acute lymphoblastic leukemia, where *CDKN2A/B* mutations have been highlighted as independent poor prognostic markers and then included in the risk stratification^26^. Further genomic studies are necessary in order to define the prognostic and predictive role of *CDKN2A/B* mutations in CCA setting, and in order to explicate the different role revealed in *IDH1*m and *IDH1*wt patients in our analysis. Another important highlight in our analysis concerns the role of *TP53* mutations in the cohort of patients treated with systemic therapy. In fact, alterations in *TP53* were highlighted to negatively impact prognosis in both *IDH1*m and *IDH1*wt patients in this setting of patients. Curiously, alterations in *KRAS* and *PIK3CA* resulted to negatively affect prognosis in *IDH1*m patients, but not in *IDH1*wt patients. As reinforce to our result, mutations in TP53 were confirmed to be negative prognostic factors in terms of both PFS and OS in patients receiving a first line therapy after adjustment for the clinical covariates known to impact on prognosis in this subset of patients. The association between mutations in both *TP53* and *KRAS* and poor prognosis is not novel for iCCA, since our data are consistent with previous reports^16,22,27,28^. Simbolo and collaborators performed a high-coverage target sequencing analysis on two groups of iCCA patients selected according to prognostic performance, and found that in the group of patients with poor prognosis (OS < 36 months) *TP53* was the most mutated gene (p = 0.011) and exclusively present in these cases. At the multivariate analysis, mutations in *TP53* have been confirmed to be independent predictors of poor prognosis^17^. The role of *TP53* in CCA was further investigated by Tian and colleagues, who performed a comprehensive genomic analysis on 66 Chinese CCA patients, thus revealing *TP53* as a suitable diagnostic and predictive biomarker in Chinese patients with CCA^24^. Association between *KRAS* mutation, perineural invasion, large bile duct type, and worse outcome after iCCA resection have also been reported^22^. In a further integrative genomic analysis, the authors found that for all patients *TP53, KRAS* and *CDKN2A* alterations predicted worse OS across all stages, even when controlling for known correlates of outcome (multifocal disease, lymph node involvement, bile duct type, periductal infiltration). In resected patients (n = 209), *TP53* mutations and *CDKN2A* deletions independently predicted shorter OS; in unresectable iCCA, *TP53, KRAS* mutations and *CDKN2A* deletions similarly predicted worse outcome^21^.

All these data are partially consistent with our results, and our research could reinforce previous insights on the genomic landscape of CCA, mainly concerning the negative prognostic role of *TP53*. On the other hand, our analysis focused on the *IDH1* mutations, thus providing, for the first time, a large sample of *IDH1*m patients.

Our research presents several limitations. Firstly, it was conducted as a retrospective investigation, thus several selection bias could be ascribed to the same nature of the study. Secondly, since several genes which resulted to significantly impact on OS at the univariate analysis belong to linked biological pathways, or to the same biological pathway, the eventual multivariate analysis would have been not informative, and for this reason has not been performed, thus reducing the powerful of the study. Moreover, in order to investigate the differences in terms of genomic landscape between *IDH1*m and *IDH1*wt CCA patients, we have recruited patients no consecutively, in order to define two large sample of patients to compare. Then, several clinic-pathological and familiar data have been excluded in our analysis, since our objective was to perform a pure genomic analysis with the definition of gene signatures able to stratify our patients. Finally, the results of our survival analysis have to be validated on an external cohort of patients. Nevertheless, it would be difficult to validate our results on an external cohort, since the most important cohort investigating CCA samples included a few proportion of *IDH1*m patients.

## Conclusion

In conclusion, we performed a comparative genomic analysis on a large sample of iCCA patients, thus highlighted several molecular differences between IDH1m and IDH1wt patients, as well as interesting highlights on the prognostic role of genomic alterations. Once validated, our results could add new pieces to the puzzle of the heterogeneous scenario of CCAs, with the ultimate goal of opening the way to further researches focused on new therapeutic strategies depending on the genomic signatures.

## Supplementary Information

Supplementary Figure 1.

Supplementary Table 1.

Supplementary Table 2.

## Data Availability

Data available on request from the authors (margherita.rimini@gmail.com).
