# Supplementary material for: Molecular profile and its clinical impact of IDH1 mutated versus IDH1 wild type intrahepatic cholangiocarcinoma
Source: Sci Rep. 2022 Nov 5;12:18775. doi: 10.1038/s41598-022-22543-z (PMC9637171; doi:10.1038/s41598-022-22543-z)
Supplement: Supplementary file 1 — Supplementary Figure 1. [file 41598_2022_22543_MOESM1_ESM.pptx]

## Slide 1
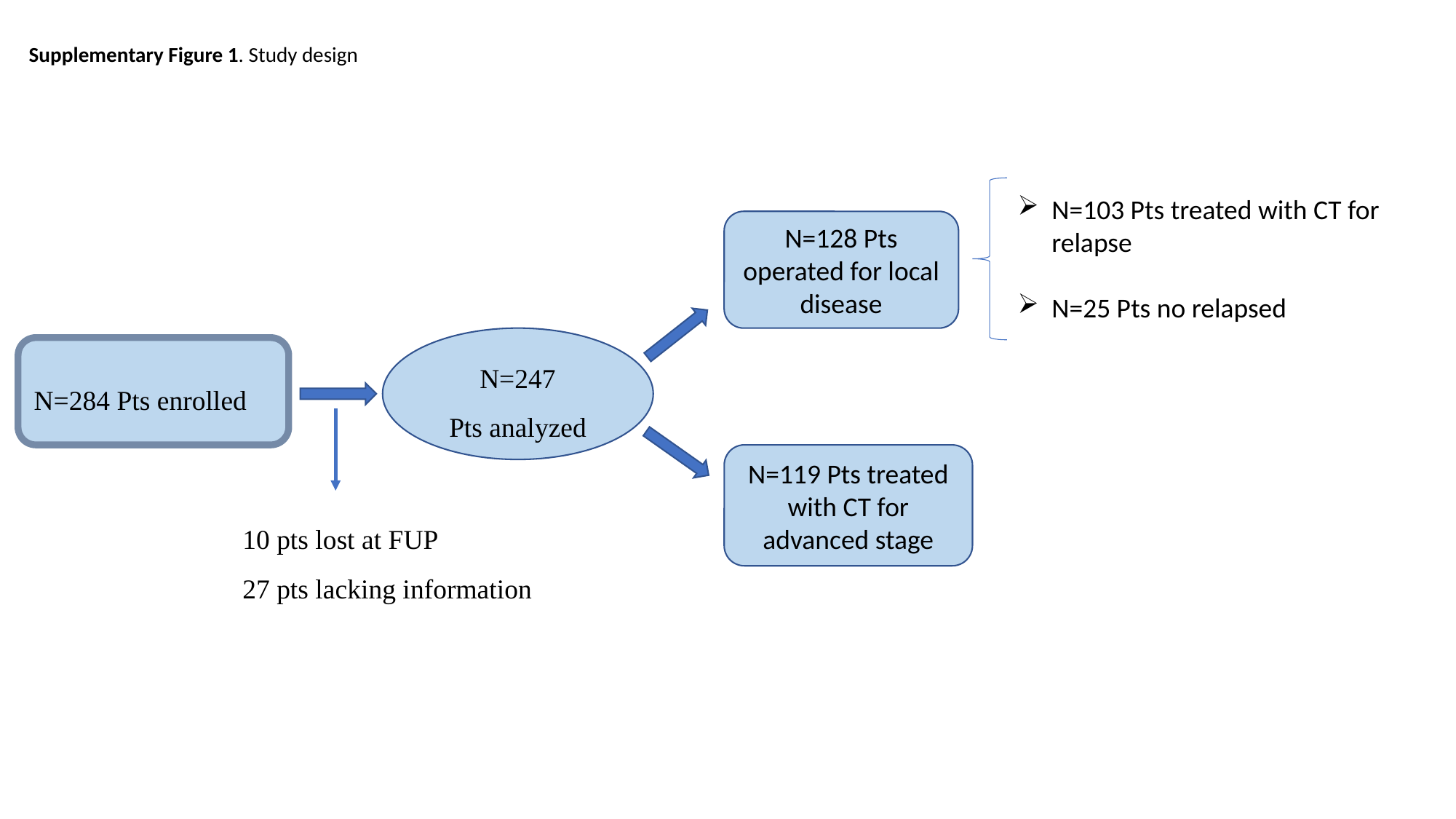

Supplementary Figure 1. Study design
N=103 Pts treated with CT for relapse
N=25 Pts no relapsed
N=128 Pts operated for local disease
N=247
Pts analyzed
N=284 Pts enrolled
N=119 Pts treated with CT for advanced stage
10 pts lost at FUP
27 pts lacking information
