# Supplementary material for: Molecular profile and its clinical impact of IDH1 mutated versus IDH1 wild type intrahepatic cholangiocarcinoma
Source: Sci Rep. 2022 Nov 5;12:18775. doi: 10.1038/s41598-022-22543-z (PMC9637171; doi:10.1038/s41598-022-22543-z)
Supplement: Supplementary file 2 — Supplementary Table 1. [file 41598_2022_22543_MOESM2_ESM.docx]

| ***GENE*** | ***TOTAL*** | | ***IDH1 MUTATED*** | | ***IDH1 WT*** | |
| --- | --- | --- | --- | --- | --- | --- |
| ***DFS from surgery*** | | | | | | |
|  | ***HR*** | ***P*** | ***HR*** | ***P*** | ***HR*** | ***P*** |
| CDKN2A | 1,9928 (1,1229-3,5365) | **0,0038** | 3,7761 (1,3692-10,4137) | **0,0001** | 1,2150 (0,6094-2,4225) | 0,5599 |
| ARID1A (WT VS M) | 1,2623 (0,7640-2,0858) | 0,3922 | 1,1487 (0,5512-2,3939) | 0,7198 | 1,3486 (0,6738-2,6993) | 0,4393 |
| CDKN2B | 2,3936 (1,1820-4,8474) | **0,0007** | 3,4647 (1,1677-10,2804) | **0,0004** | 1,4192 (0,5315-3,7893) | 0,4208 |
| PBRM1 (WT VS M) | 1,2209 (0,7366-2,0237) | 0,4630 | 1,2437 (0,5893-2,6247) | 0,5872 | 1,0692 (0,5246-2,1790) | 0,8568 |
| KRAS/NRAS | 1,3663 (0,6468-2,8864) | 0,3531 | 0,9885 (0,2389-4,0896) | 0,9873 | 1,4146 (0,5988-3,3418) | 0,3691 |
| BAP1 (WT Vs M) | 1,0043 (0,6016-1,6765) | 0,9869 | 1,6738 (0,7844-3,5717) | 0,2455 | 0,7776 (0,3939-1,5353) | 0,4394 |
| TP53 (WT VS M) | 1,2728 (0,7457-2,1723) | 0,4107 | 0,6064 (0,1396-2,6348) | 0,4032 | 1,5750 (0,8554-2,8999) | 0,1836 |
| FGFR2 | 1,0342 (0,5679-1,8835) | 0,9115 | 0,8163 (0,3735-1,7843) | 0,6304 | 1,3680 (0,5198-3,6001) | 0,4717 |
| BRCA2 (WT VS M) | 1,0587 (0,5189-2,1599) | 0,8781 | - | 0,0882 | 0,8050 (0,3547-1,8273) | 0,5756 |
| PIK3CA (WT VS M) | 1,6054 (0,7096-3,6322) | 0,3513 | 1,9739 (0,6754-5,7687) | 0,3412 | 1,4541 (0,4392-4,8148) | 0,6023 |
| ATM | 1,3223 (0,5172-3,3808) | 0,5086 | 2,0559 (0,1238-34,1335) | 0,4679 | 1,2301 (0,4490-3,3705) | 0,6610 |
| MTAP | 1,9825 (0,4985-7,8848) | 0,1735 | 8,2892 (0,1673-410,6821) | **0,0005** | 1,1366 (0,2530-5,1058) | 0,8593 |
| MAP3K1 (WT VS M) | 1,0364 (0,4571-2,3499) | 0,9327 | 0,3683 (0,01475-9,1966) | 0,3049 | 1,3210 (0,5736-3,0426) | 0,5549 |
| ***OS from surgery*** | | | | | | |
| CDKN2A | 1.9341 (0,9594-3.8993) | **0,0291** | 3,1962 (1,0075-10,1400) | **0,0096** | 1,0477 (0,4600-2,3861) | 0,9107 |
| ARID1A (WT Vs M) | 1,5121 (0,7487-3,0536) | 0,3094 | 1,0186 (0,3372-3,0771) | 0,9741 | 1,8527 (0,7126-4,8169) | 0,3042 |
| CDKN2B | 2,0608 (0,9099-4,6676) | 0,0272 | 3,1962 (1,0075-10,1400) | **0,0096** | 1,0777 (0,3642-3,1888) | 0,8895 |
| PBRM1 (WT Vs M) | 1,5136 (0,7726-2,9649) | **0,2821** | 6,6111 (2,3628-18,4978) | **0,0339** | 0,8678 (0,3586-2,1004) | 0,7437 |
| KRAS/NRAS | 1,1849 (0,4392-3,1973) | 0,7195 | 1,1854 (0,1344-10,4524) | 0,8686 | 1,1414 (0,3768-3,4577) | 0,8059 |
| BAP1 (WT Vs M) | 1,0444 (0,5239-2,0823) | 0,9029 | 1,6532 (0,5676-4,8151) | 0,4218 | 0,8641 (0,3566-2,0936) | 0,7362 |
| TP53 | 1,1930 (0,5521-2,5779) | 0,6344 | 5,3819 (0,2437-118,8673) | **0,0121** | 0,8019 (0,3578-1,7973) | 0,6102 |
| FGFR2 | 1,3706 (0,5861-3,2056) | 0,4147 | 1,2840 (0,3878-4,2516) | 0,6584 | 2,1795 (0,5154-9,2169) | 0,1379 |
| BRCA2 | 2,1258 (0,7230-6,2503) | 0,0595 | - | - | 1,9774 (0,6897-5,6697) | 0,1092 |
| PIK3CA (WT Vs M) | 1,3569 (0,3949-4,6627) | 0,6716 | 0,6878 (0,1243-3,8066) | 0,6157 | - | 0,5045 |
| ATM | 2,5548 (0,6388-10,2175) | **0,0398** | - | 0,7763 | 2,0165 (0,5781-7,0338) | 0,1453 |
| MTAP | 2,9023 (0,2780-30,3002) | 0,1223 | 40,1765 (0,0002254-7162536,3323) | **< 0,0001** | 1,2951 (0,1362-12,3133) | 0,7989 |
| MAP3K1 | 1,4753 (0,3670-5,9312) | 0,5119 | 4,2267 (0,07973-224,0632) | 0,1272 | 1,0015 (0,2379-4,2154) | 0,9984 |

**Supplementary Table 1**. Univariate analysis for DFS and OS from surgery according to the single gene alteration in IDH1m and IDh1wt patients.
