# Supplementary material for: Molecular profile and its clinical impact of IDH1 mutated versus IDH1 wild type intrahepatic cholangiocarcinoma
Source: Sci Rep. 2022 Nov 5;12:18775. doi: 10.1038/s41598-022-22543-z (PMC9637171; doi:10.1038/s41598-022-22543-z)
Supplement: Supplementary file 3 — Supplementary Table 2. [file 41598_2022_22543_MOESM3_ESM.docx]

| ***GENE*** | ***TOTAL*** | | ***IDH1 MUTATED*** | | ***IDH1 WT*** | |
| --- | --- | --- | --- | --- | --- | --- |
| ***PFS from I line therapy*** | | | | | | |
|  | ***HR*** | ***P*** | ***HR*** | ***P*** | ***HR*** | ***P*** |
| CDKN2A | 1,2282 (0,8688-1,7364) | 0,2446 | 1,0946 (0,6942-1,7259) | 0,6921 | 1,3002 (0,7741-2,1836) | 0,3211 |
| ARID1A | 1,0104 (0,6719-1,5194) | 0,9603 | 0,6728 (0,4061-1,1146) | 0,1711 | 1,7518 (0,8946-3,4304) | 0,1020 |
| CDKN2B | 1,7922 (1,1504-2,7921) | **0,0099** | 1,5404 (0,8924-2,6590) | 0,0764 | 1,9284 (0,9242-4,0238) | 0,0801 |
| PBRM1 (WT VS M) | 1,2199 (0,8302-1,7926) | 0,3114 | 1,0912 (0,6167-1,9308) | 0,7711 | 1,3607 (0,8024-2,3072) | 0,2530 |
| KRAS/NRAS | 2,0436 (1,2430-3,3596) | **0,0048** | 2,0640 (0,9447-4,5094) | **0,0136** | 1,6759 (0,8795-3,1933) | 0,1165 |
| BAP1 (WT Vs M) | 1,1800 (0,7958-1,7496) | 0,4103 | 1,0727 (0,5654-2,0351) | 0,8344 | 1,2453 (0,7456-2,0801) | 0,4019 |
| TP53 | 1,6933 (1,0098-2,8396) | **0,0458** | 2,0483 (0,8039-5,2189) | **0,0377** | 1,3408 (0,7185-2,5021) | 0,3568 |
| FGFR2 | 1,2476 (0,7971-1,9527) | 0,3331 | 1,1856 (0,6200-2,2674) | 0,5829 | 1,2796 (0,6927-2,3637) | 0,4310 |
| BRCA2 | 1,0455 (0,5841-1,8714) | 0,8810 | 8,8309 (0,1489-523,6687) | **0,0002** | 0,8459 (0,4602-1,5546) | 0,5899 |
| PIK3CA (WT VS M) | 1,2604 (0,7042-2,2558) | 0,4358 | 1,5581 (0,7825-3,1026) | 0,2902 | 0,8438 (0,2826-2,5190) | 0,7608 |
| ATM | 1,9669 (0,9825-3,9377) | 0,0561 | 13,8866 (0,3867-498,6739) | **< 0,0001** | 1,1232 (0,5562-2,2684) | 0,7459 |
| MTAP | 2,7863 (1,2289-6,3178) | **0,0142** | 1,7269 (0,5427-5,4958) | 0,2295 | 3,4025 (1,1063-10,4646) | **0,0327** |
| MAP3K1 | 1,3916 (0,7287-2,6576) | 0,3168 | 3,1970 (0,4281-23,8754) | 0,0364 | 1,0881 (0,5442-2,1754) | 0,8113 |
| ***OS from I line therapy*** | | | | | | |
| CDKN2A | 1.2564 (0.8446-1.8688) | 0.2385 | 1.1897 (0.6857- 2.0640) | 0.5367 | 1.3803 (0.7534-2.5286) | 0.2645 |
| ARID1A | 1.1837 (0.7441-1.8829) | 0.4532 | 1.1983 (0.6485-2.2140) | 0.5636 | 1.1690 (0.5669-2.4106) | 0.6561 |
| CDKN2B | 1.5956 (0.9361-2.7198) | **0.0422** | 1.8178 (0.9181-3.5991) | 0.0864 | 1.4500 (0.6116-3.4373) | 0.3301 |
| PBRM1 | 1.2115 (0.7467-1.9655) | 0.4079 | 0.9806 (0.4970-1.9351) | 0.9550 | 1.5309 (0.7539-3.1089) | 0.1789 |
| KRAS/NRAS | 1.1044 (0.6569-1.8566) | 0.6978 | 1.7616 (0.7505-4.1349) | 0.1934 | 0.9125 (0.4549-1.8303) | 0.8022 |
| BAP1 (WT Vs M) | 15008 (0.9382-2.4007) | 0.1388 | 1.5176 (0.7473-3.0821) | 0.2485 | 1.2905 (0.6655-2.5027) | 0.4845 |
| TP53 | 1.9368 (1.0316-3.6363) | **0.0072** | 4.3924 (1.3393-14.4051) | **0.0146** | 1.9683 (0.8827-4.3893) | **0.0355** |
| FGFR2 | 1.1698 (0.7046-1.9423) | 0.5219 | 1.0340 (0.4844-2.2075) | 0.9311 | 1.4187 (0.6940-2.9003) | 0.2841 |
| BRCA2 (WT VS M) | 1.2197 (0.6525-2.2798) | 0.5662 | 1.1552 (0.1811-7.3692) | 0.8787 | 1.0490 (0.5006-2.1980) | 0.9007 |
| PIK3CA | 2.0240 (0.8727-4.6941) | **0.0230** | 2.6732 (1.0884-6.5657) | **0.0320** | 1.0574 (0.1385-8.0731) | 0.9559 |
| ATM | 1.5779 (0.7172-3.4713) | 0.1638 | 3.7969 (0.6628-21.7505) | 0.1341 | 1.6149 (0.6185-4.2163) | 0.2330 |
| MTAP | 1.7391 (0.6515-4.6424) | 0.1503 | 2.2434 (0.5858-8.5919) | 0.2383 | 1.5928 (0.3776-6.7188) | 0.4294 |
| MAP3K1 | 0.8389 (0.2322-3.0301) | 0.8051 | 1.5883 (0.3927-6.4232) | 0.5163 | 1.3923 (0.5275-3.6752) | 0.4431 |
| IDH1 | 1.3330 (0.9304-1.9098) | 0.1179 |  |  |  |  |

**Supplementary Table 2**. Univariate analysis for PFS and OS from the start of the first line therapy according to the single gene alteration in IDH1m and IDh1wt patients.
